# Improvement in depressive symptoms in a patient with severe and enduring anorexia nervosa and comorbid major depressive disorder using psychotherapy-assisted IV ketamine : a case report

**DOI:** 10.1186/s40337-024-01039-3

**Published:** 2024-06-12

**Authors:** Amanda Timek, Catherine Daniels-Brady, Stephen Ferrando

**Affiliations:** Westchester Medical Center, 100 Woods Road, 10595 Valhalla, NY USA

**Keywords:** Severe and enduring anorexia nervosa, Major depressive disorder, Treatment-resistant depression, Ketamine-assisted psychotherapy, Case report

## Abstract

**Background:**

Anorexia nervosa is a life-threatening psychiatric illness with a high mortality rate and limited treatment options. This illness is frequently comorbid with major depressive disorder, leading to additional obstacles in patient quality of life, and increasing the mortality rate further due to risk of suicide. Ketamine, a competitive N-methyl-D-aspartate receptor antagonist, has been shown to be beneficial in depression given its effects on neuroplasticity. There are few cases in the literature describing ketamine use in patients with eating disorders, and even fewer that describe psychotherapy-assisted ketamine use in this patient population. We present the case of a 33-year-old woman with a history of severe and enduring anorexia nervosa and comorbid major depressive disorder who we treated safely with ketamine-assisted psychotherapy using intravenous ketamine in a general hospital setting.

**Case presentation:**

Our patient is a 33-year-old woman with past psychiatric history of severe and enduring anorexia nervosa and major depressive disorder with comorbid psychiatric and medical conditions who presented to the hospital due to malnutrition. She had an extensive psychiatric history as well as multiple medical hospitalizations due to her eating disorder. She had tried numerous psychiatric treatments, including antidepressants, mood stabilizers, antipsychotics, electroconvulsive therapy, and multiple types of therapies without significant improvement in symptoms. She agreed to try ketamine for treatment-resistant depression and received it intravenously for seven sessions in a closely monitored setting, and simultaneously engaged in acceptance and commitment therapy during sessions. She demonstrated increased cognitive flexibility, disappearance of suicidal ideation, and reduction in Beck Depression Inventory Scores.

**Conclusions:**

Our case is unique in that it demonstrates the successful usage of ketamine-assisted psychotherapy in a hospital setting with severe and enduring anorexia nervosa and comorbid major depressive disorder. Her body mass index was profoundly low at 13, whereas the lowest documented in the literature was 16.9. This case shows that ketamine-assisted psychotherapy may be a promising treatment modality for patients with anorexia nervosa with co-morbid depression who have failed other interventions.

## Introduction

Anorexia Nervosa (AN) is a psychiatric disorder with a high mortality rate. Symptoms include significantly low body weight, food restriction, disturbance in body image, intense fear of weight gain or behaviors that interfere with weight gain [1]. Patients with AN have over twice the mortality rate of those without. These deaths appear to be attributable both to the medical sequelae of anorexia nervosa, and to suicide [2]. The mainstays of treatment for AN are psychotherapy and nutritional intervention. In spite of the available treatment options, over 20% of patients develop a chronic course [3]. These patients are said to have severe and enduring AN (SE-AN). While the exact criteria for SE-AN are not precisely defined, most literature describes elements of unremitting AN symptoms (low BMI), long duration (over 7 years), and treatment resistance (failure of multiple high-quality treatment interventions) [4]. There is frequent comorbidity between AN and Major Depressive Disorder (MDD) and standard antidepressant treatments do not appear to robustly improve clinical course in this patient population [5]; therefore consideration should be given to new types of antidepressant therapies. One such treatment that has garnered interest in recent years for the treatment of MDD is ketamine.

Ketamine is a non-competitive N-methyl-D-aspartate (NMDA) receptor antagonist, and has been shown to be efficacious in treatment-resistant depression (TRD). Esketamine, the s-enantiomer of ketamine, has been FDA-approved to treat TRD. A possible mechanism for the antidepressant effect of ketamine is neuroplastic changes via activation of brain-derived neurotrophic factor and its receptor tropomycin receptor kinase B signaling cascade and their downstream changes, especially in the medial prefrontal cortex. Chronic stress in depression-related behavior has been shown to eliminate postsynaptic dendritic spines on prefrontal projection neurons. Antidepressant-dose ketamine may reverse these effects by selectively rescuing eliminated spines [6]. Ketamine may also have beneficial effects in eating disorders. Since NMDA receptors are responsible for long-term potentiation, and are involved in memory and recall in the hippocampus, ketamine may prevent reinforcement of compulsive behavior; this compulsive behavior is one of the factors that perpetuate anorexia nervosa [7]. Studies involving mice have demonstrated favorable effects of ketamine on activity-based anorexia, in which a single dose of ketamine increased food intake and weight gain and reduced anxiety-like behavior, with long-lasting effects [8].

The literature on ketamine use for eating disorders is limited to case reports and case series [7, 9–13]. Even fewer reports exist of the utilization of psychotherapy during ketamine sessions. The first case report to detail ketamine-assisted psychotherapy in an eating disorder patient was in 2021, and demonstrated prolonged reduction of eating disorder symptoms after using intravenous ketamine in a patient with refractory bulimia nervosa [14]. A case series examined improved depression and anxiety in a series of patients with various eating disorders utilizing group-based ketamine-assisted psychotherapy with intramuscular ketamine [15]. Another case series described ketamine-assisted psychotherapy with sublingual followed by intramuscular ketamine in adolescents with various psychiatric diagnoses, including anorexia nervosa and unspecified eating disorder with binging/purging/restricting, which showed symptomatic and functional improvements [16].

To our knowledge, we are the first to present a case of intravenous (IV) ketamine-assisted psychotherapy to a patient with anorexia nervosa with very low weight. The aforementioned cases describe patients with normal BMI or underweight; the lowest described BMI in previous literature is 16.9. Given the medical fragility of patients with severe underweight, we wanted to present a case that demonstrates the safe use of intravenous ketamine in this population. We also wanted to describe the utility and feasibility of utilizing ketamine-assisted therapy in a patient so acutely ill as to require inpatient medical hospitalization. We therefore present the case of a 33-year-old woman with a history of severe and enduring anorexia nervosa and comorbid major depressive disorder, who we treated safely with ketamine-assisted psychotherapy using IV ketamine in a general hospital setting.

## Case presentation

Our patient “Ms. S.” is a 33-year-old woman with a history of severe and enduring anorexia nervosa (SE-AN), gastroparesis, epilepsy, major depressive disorder, generalized anxiety disorder (GAD), and post-traumatic stress disorder (PTSD) who was admitted to the hospital with severe malnutrition. At the time of her admission, she had a body mass index (BMI) of 13 kg/m^2^(indicating extreme underweight status), and had restricted her caloric intake to 100 kcal per day due to stomach pain and fear of losing control while eating.

Ms. S has an extensive psychiatric history. Her anorexia nervosa symptoms started at age 13. She attended specialized treatment for anorexia nervosa starting in her teen years. She had modest treatment success at several points in time, notably during her early twenties, a period of time during which she completed schooling and pursued a professional career. In her mid- to late- twenties she again became symptomatic of her eating disorder and received intensive and highly specialized treatments for eating disorders, including several residential eating disorder treatment programs. She had a history of medical sequelae of severe malnutrition, including gastroparesis and slow-transit constipation. She has required admission to medical units numerous times for monitored refeeding, and has developed electrolyte abnormalities and extreme bradycardia. During several of these admissions she was found to engage in extreme avoidance of the consumption of calories. She had a history of concealing food to falsely elevate calorie counts, and tampered with medical devices that delivered calories, such as naso-gastric tube connections. At times, she would have episodes of behavioral dysregulation, including removing therapeutic lines, attempting impulsively to leave the hospital against medical advice.

In addition to the SE-AN, Ms. S has suffered from depression and anxiety since her late teenage years. She has had numerous psychiatric hospitalizations and several prior suicide attempts. She has a history of non-suicidal self-injurious behavior. Her last suicide attempt was by wrist cutting, requiring sutures by a hand surgeon, approximately two months prior to this admission. For treatment of her depression and anxiety she has failed numerous medication trials. The details regarding duration and dosage of these treatments was not accessible to us at the time of our treatment of Ms. S. She had many antidepressant and augmentation trials over a twenty- year period from all the major classes of antidepressant medications without significant clinical response. She could not tolerate selective serotonin reuptake inhibitors (SSRIs), serotonin and norepinephrine reuptake inhibitors (SNRIs) and Buspirone due to reported side effects of agitation, and also failed trials of tricyclic antidepressants (TCAs). She was treated with typical and atypical antipsychotics, anti-epileptic mood stabilizers, and psychostimulants, which were not efficacious. She failed three previous full courses of electroconvulsive therapy. The patient had had one prior trial of ketamine more than ten years prior; however, the details of this trial are not known and it seems she did not finish a full ketamine course. She has a history of trauma, including sexual trauma in early adulthood. She has engaged in psychotherapy both during hospitalizations and as an outpatient, in various modalities including cognitive-behavioral therapy, dialectical-behavior therapy, and acceptance and commitment therapy.

Ms. S has a history of numerous medical hospitalizations due to her malnourishment. She had been discharged from our hospital one month prior to this presentation, but had been unable to maintain the weight she had gained during the previous hospitalization. At the time of this hospital admission, she was afebrile, with bradycardia (52 beats per minute) and hypotension (blood pressure 88/45). Labs were significant for leukopenia (white blood cell count 2.8 k/mm^3^) and anemia (Hemoglobin 10.7 g/dL); electrolytes, liver function tests and coagulation studies were normal. Her home medications were restarted, which included Clonazepam 1 mg in the morning and 1.5 mg in the evening, Lorazepam 2 mg three times daily with meals, Metoclopramide 10 mg three times a day with meals, Pyridostigmine 90 mg three times daily, Quetiapine 200 mg two times daily, Trazodone 100 mg three times daily plus 300 mg at bedtime, and Gabapentin 600 mg three times daily. Chest x-ray showed clear lungs and electrocardiogram (EKG) showed sinus bradycardia. A few days after admission, the patient was transferred to the medical intensive care unit for management of hypotension; she was given a 1-liter bolus of normal saline and 1-liter of lactated ringers; she was briefly treated with Norepinephrine, and was then transferred back to the floors after blood pressure was stabilized.

The psychiatry team was consulted upon admission for assistance with managing Ms. S’s disordered eating behaviors and mood and anxiety disorder symptoms; psychiatry followed her throughout her admission. On assessment, Ms. S was quite symptomatic of SE-AN with severe and entrenched eating disorder cognitions: panic at the thought of consuming calories, preoccupied with thoughts of food and rigid rules around eating in order to influence weight, intense fear of losing control over eating, shame when seeing her body in the mirror. In response to her fears about eating and weight, she fixated on her anxiolytic medications and attempted to negotiate with providers for higher doses of her medications; she recognized that in the past higher doses had yielded sedation but not true anxiolysis. She complained of severe depression, in spite of a robust regimen of antidepressant medications. Themes of her depression included sadness, loss of interest, feeling slowed down, seeing self as a failure and causing problems for others, and self-dislike. She had suicidal ideation without acute intent or plan. She felt absolutely unmotivated to challenge her anxious and eating-disorder based cognitions - the psychiatric consultants formulated that her depression created such profound symptoms that rendered behavioral treatment of her SE-AN and GAD prohibitively difficult. She scored 42 on the Beck Depression Inventory (BDI) and 26 on the Quick Inventory of Depressive Symptomatology. Since the patient had tried multiple medications and interventions for her MDD without adequate therapeutic benefit, she was considered to have TRD and alternative options were explored.

The patient was willing to try ketamine to address her depression with the hope that it would ameliorate her distress and enhance collaboration in the care of her eating disorder. She expressed hope that ketamine could help with her fear of nutrition and calories. She understood that ketamine is an off-label treatment for treatment-resistant major depressive disorder, and that there could be no guarantee of benefit. She reasoned that extensive on-label treatments over many years had not helped her substantially, and therefore she was willing to undergo this off-label treatment. Extensive discussion of possible risks was undertaken; she asked appropriate questions and evidenced understanding. Based on her reasoning, understanding, and appreciation of her condition she was considered capable of consenting to ketamine infusion for depression. She then provided written informed consent to undergo ketamine-assisted psychotherapy. She was medically cleared for ketamine infusion therapy by her primary internal medicine team.

Ms. S received seven ketamine infusions over 3 weeks. She initially received 0.5 mg/kg IV ketamine over 40 min; the dose was increased up to 1 mg/kg based on response and tolerability. She received 0.5 mg/kg for the first infusion, 0.75 mg/kg for the second through 5th infusions, and 1 mg/kg for the sixth and seventh infusions. The infusions were performed in the operating room staging area, with an anesthesiologist available throughout the infusion. Her heart rate, respiratory rate, and oxygen saturation were monitored continuously, and her blood pressure was measured every 15 minutes. She was assessed throughout treatment for adverse effects. Her vital signs were monitored every 30 minutes for 2 hours after the infusion, and she was kept on constant cardiac telemetry while on the medical-surgical floors.

Overall, she tolerated the ketamine infusions well. During two sessions she experienced nausea and received ondansetron 4 mg IV within two hours of the infusion, with good effect. She experienced mild dissociation during four infusions and lorazepam 1 mg IV was given afterward for the associated anxiety, with good effect. After her third infusion, she had a brief two-second episode of tachyarrhythmia (heart rate of 132) on telemetry which spontaneously resolved, and was deemed by the medical team to not require intervention. Subsequent continuous telemetry monitoring was unremarkable. Additionally, she experienced mild sedation and a drop in her blood oxygen level during treatment 6, when the dose was increased to 1 mg/kg. She briefly required oxygen, 2 liters per minute via nasal cannula, after which her oxygen saturation returned to normal and she was weaned from nasal cannula oxygen to room air. This did not recur on treatment 7 when she received the same dose. Although the patient tolerated the infusions quite well, the potential for adverse effects underline the importance of continuous monitoring for these patients during ketamine infusions, and the necessity of working as an interdisciplinary team.

**Fig. 1:**
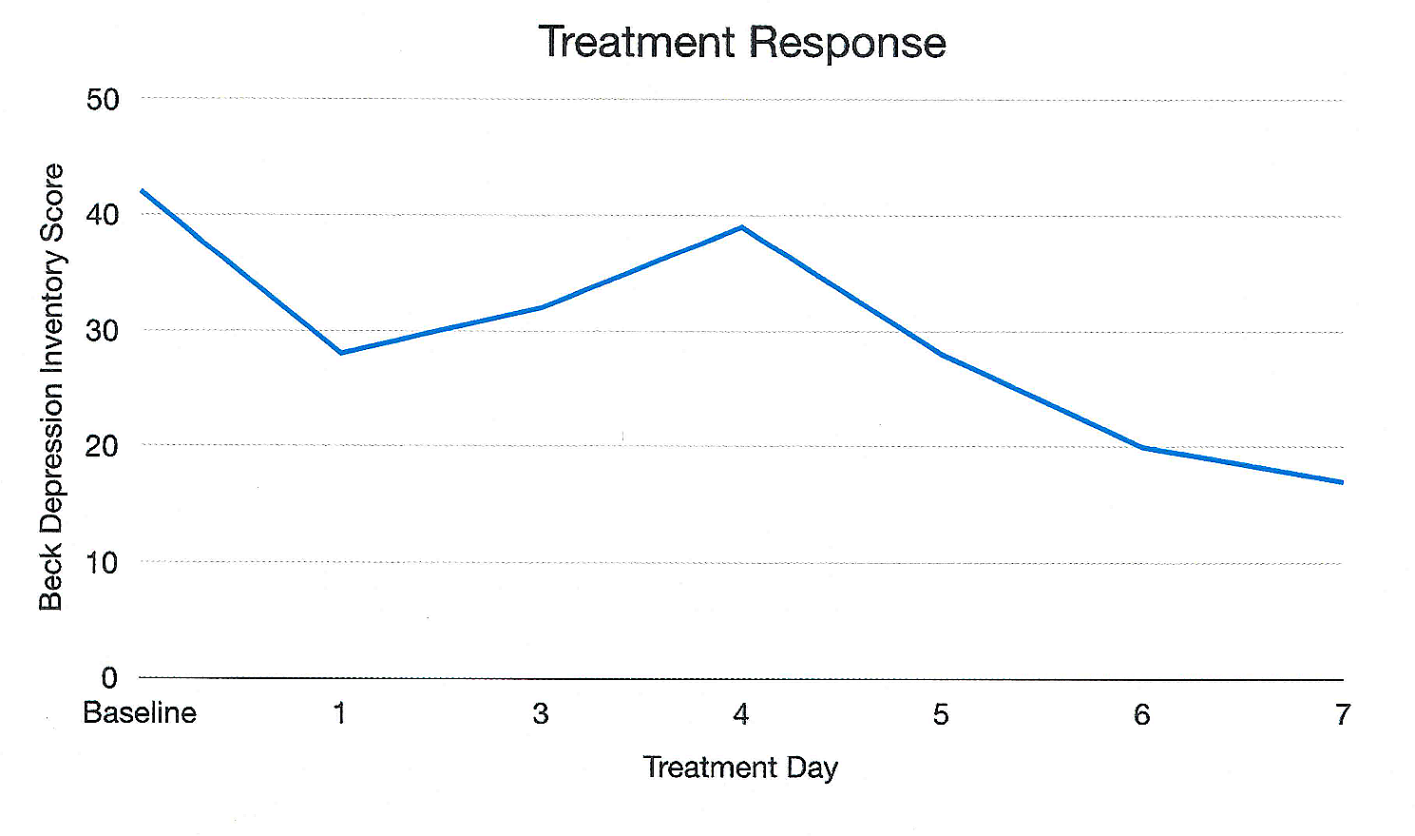
Graph of patient’s Beck Depression Inventory scores. Initial score before ketamine-assisted psychotherapy was 42. Additional scores were collected after each session. Patient experienced an elevated score around treatment day three when she transitioned from TPN to Ensure. She had a further decrease in her score with continued ketamine infusions. Her score at discharge was 17.

During each session, Ms. S engaged in acceptance and commitment therapy (ACT) with the attending psychiatrist (CD-B). ACT is a “third-wave” psychotherapy based on the premise that unpleasant emotions are a fundamental part of human life; and that accepting rather than eliminating these experiences reduces the suffering caused by them. In ACT, patients are taught skills for dealing with their internal experiences that increase their cognitive flexibility [17]. During ketamine-assisted psychotherapy sessions, Ms. S was observed to be open to sharing her thoughts and feelings, thought about her future dreams and stated goals, expressed that she could give up rules and control in regards to her eating, could process her thoughts and feelings with more flexibility, allowed herself to eat more calorie-dense foods than usual, and ate a scone with the provider at the last session. BDI Scores were obtained after each session (see Fig. 1).

After an initial 35% drop in her BDI, there was some variability in her depression severity scores. Of note, on treatment day three, her nutritional intake was transitioned from total parenteral nutrition (TPN) to an oral nutritional supplement (Ensure). This caused her a great deal of distress, and we believe that this is reflected in her elevated BDI score. However, with continued ketamine infusions, the patient’s final BDI Score had decreased to 17. The patient no longer had suicidal ideation at the end of the treatment.

The patient was transitioned from TPN to oral nutritional supplement and then to a regular oral diet. On the day of discharge, her BMI was 14.8, representing a nearly 4-kilogram weight gain over 4 weeks. She was given instructions to follow up with gastroenterology and psychiatry as well as a ketamine clinic as an outpatient, with a recommendation to pursue maintenance outpatient ketamine infusions.

The patient was contacted in order to provide consent to publish this case report, and she provided an update. She had been unable to follow up with an outpatient ketamine clinic. She has been hospitalized twice since the events of this case report for continued stomach pain, related to chronic gastroparesis. She does not report any lingering adverse effects from her ketamine-assisted psychotherapy. She has been doing well psychologically, denies symptoms of depression, and had reportedly gained another four kilos. She expresses further interest in trying ketamine again given its positive effects.

## Discussion and conclusions

This case demonstrates the successful usage of ketamine-assisted psychotherapy in a hospital setting with a patient with severe underweight due to severe and enduring anorexia nervosa, and comorbid treatment-resistant depression. Given Ms. S’s recalcitrant clinical course, with multiple suicide attempts, severe impairment in social and occupational functioning, and TRD which rendered her eating disorder difficult to treat, ketamine-assisted psychotherapy was felt to be indicated.

She was able to demonstrate understanding of the treatment’s indications and risks, and to demonstrate understanding that she might not in fact benefit from the ketamine treatment. The team discussed the treatment rationale and limitations extensively with the patient and her family. Because of her significant symptom burden and treatment resistance, this treatment was undertaken within a “compassionate use” framework; indeed she met criteria for compassionate use based on Food and Drug Administration (FDA) criteria, namely her serious condition with no satisfactory alternative treatment, and the potential benefit [18]. This course of action was chosen by the treatment team for two compelling reasons - both the direct antidepressant effects of ketamine in light of her significantly high depression scores and due to the strong cognitive and behavioral symptoms that perpetuated her condition. In particular, we felt that psychotherapy aimed at reducing the patient’s rigidity and increasing her psychological flexibility could be possible through use of ketamine; previous attempts at these treatment goals were unsuccessful.

Overall, the patient tolerated the sessions well, with an observable decrease in her BDI Score and disappearance of suicidal ideation. In addition, the patient was engaged in ketamine-assisted psychotherapy during her infusions, which appears to have resulted in increased cognitive flexibility and increased willingness to change thinking patterns about her eating. Particularly, she was able to identify a core belief that she is not sick enough to deserve help, and that this belief led her to be more entrenched in her disordered eating behaviors, as a way of continuing to receive help. Additionally, she was able to be more present with her emotions of sadness and fear, rather than making maladaptive efforts to avoid those feelings. She was able to identify her life values, and ways in which she could work toward them. She took the committed action of eating more calorie-dense foods. See Table 1 for additional details. One limitation, however, is that we did not officially assess her changes in cognitive flexibility and thought patterns, which would be a useful outcome to measure in future cases.

**Table 1:** Examples of ACT processes during ketamine-assisted psychotherapy

ACT Process	Examples from KAP with Ms. S
Contact with present moment	Listening to music Holding mother’s hand
Self as context	“I’m not sick enough to deserve to be helped” Able to observe this thought and explore how it affects her behaviors
Defusion	Identified thoughts such as “not sick enough” to warrant help as being a product of her mind, rather than representing reality
Acceptance	Expressed feelings of fear and sadness without efforts to avoid (usually attempts to control/bargain in response to those feelings)
Values	Engaged with her values – namely being able to help others
Committed action	Ate a scone with the treating psychiatrist

Table 1: This table depicts examples of ACT processes that Ms. S. engaged in during her ketamine-assisted psychotherapy. The left column depicts these processes and include contact with present moment, self as context, defusion, acceptance, values and committed action. The right column depicts examples of each of these processes.

Our case is unique from other cases using ketamine-assisted psychotherapy in a few ways. While other reports have described KAP in other eating disorders [14, 16], ours describes a patient with SE-AN. One case series examined ketamine use with group psychotherapy whereas our patient underwent individual therapy [15]. Our patient’s BMI was also profoundly lower than other patients described in the literature. The lowest documented BMI in the relevant literature of ketamine use with eating disorders was 16.9 whereas our patient had a BMI of 13. In spite of this low BMI, ketamine infusions were given without severe medical adverse events, and with clear therapeutic benefit.

Proposed mechanisms of ketamine-assisted psychotherapy for depression and other disorders include induction of a psychedelic-like dissociative and transpersonal experience, reduced defensiveness and enhanced engagement with the therapist. Neuroplasticity also may play a role, particularly in accessing and restructuring traumatic memories, fear extinction, and increased executive control and learning. Ketamine may be used to augment the effects of psychotherapy through enhanced conscious awareness [19]. It is not precisely clear how ketamine-assisted psychotherapy works for eating disorders, however, there was an observed reduced defensiveness, increased cognitive flexibility and improved reflection ability for our patient.

This case is important because it illustrates the potential benefit of ketamine-assisted psychotherapy for treatment of patients with anorexia nervosa with co-morbid depression who have failed other interventions. SE-AN and TRD are highly debilitating and morbid conditions and new modalities are needed to treat them.

## Data Availability

No datasets were generated or analysed during the current study.

## Declarations

### Author details

CD-B is an attending psychiatrist who engaged the patient in acceptance and commitment therapy during ketamine infusions.

## Abbreviations

ACT: Acceptance and Commitment Therapy
AN: Anorexia nervosa
BDI: Beck Depression Inventory
BMI: Body mass index
EKG: Electrocardiogram
FDA: Food and Drug Administration
GAD: Generalized anxiety disorder
IV: Intravenous
MDD: Major depressive disorder
NMDA: Non-competitive N-methyl-D-aspartate
PTSD: Post-traumatic stress disorder
SE-AN: Severe and enduring anorexia nervosa
SNRI: Selective serotonin and norepinephrine reuptake inhibitor
SSRI: Selective serotonin reuptake inhibitor
TCA: Tricyclic antidepressant
TPN: Total parenteral nutrition
TRD: Treatment-resistant depression
